# miR-379 Regulates Cyclin B1 Expression and Is Decreased in Breast Cancer

**DOI:** 10.1371/journal.pone.0068753

**Published:** 2013-07-10

**Authors:** Sonja Khan, Cathy L. Brougham, James Ryan, Arisha Sahrudin, Gregory O’Neill, Deirdre Wall, Catherine Curran, John Newell, Michael J. Kerin, Roisin M. Dwyer

**Affiliations:** 1 Discipline of Surgery, School of Medicine, National University of Ireland, Galway, Galway, Ireland; 2 HRB Clinical Research Facility and School of Mathematics, Statistics and Applied Mathematics, National University of Ireland, Galway, Galway, Ireland; Bambino Gesu' Children Hospital, Italy

## Abstract

MicroRNAs are small non-coding RNA molecules that control gene expression post-transcriptionally, and are known to be altered in many diseases including breast cancer. The aim of this study was to determine the relevance of miR-379 in breast cancer. miR-379 expression was quantified in clinical samples including tissues from breast cancer patients (n=103), healthy controls (n=30) and patients with benign breast disease (n=35). The level of miR-379 and its putative target Cyclin B1 were investigated on all breast tissue specimens by RQ-PCR. Potential relationships with gene expression and patient clinicopathological details were also determined. The effect of miR-379 on Cyclin B1 protein expression and function was investigated using western blot, immunohistochemistry and proliferation assays respectively. Finally, the levels of circulating miR-379 were determined in whole blood from patients with breast cancer (n=40) and healthy controls (n=34). The level of miR-379 expression was significantly decreased in breast cancer (Mean(SEM) 1.9 (0.09) Log_10_ Relative Quantity (RQ)) compared to normal breast tissues (2.6 (0.16) Log_10_ RQ, p<0.01). miR-379 was also found to decrease significantly with increasing tumour stage. A significant negative correlation was determined between miR-379 and Cyclin B1 (r=-0.31, p<0.001). Functional assays revealed reduced proliferation (p<0.05) and decreased Cyclin B1 protein levels following transfection of breast cancer cells with miR-379. Circulating miR-379 was not significantly dysregulated in patients with breast cancer compared to healthy controls (p=0.42). This data presents miR-379 as a novel regulator of Cyclin B1 expression, with significant loss of the miRNA observed in breast tumours.

## Introduction

MicoRNAs (miRNAs) are a class of small (19-25 nucleotides), non-coding RNA molecules which can be found in all eukaryotic cells, and repeatedly act to inhibit gene expression post-transcriptionally. They play key roles in regulation of gene expression by complementarily binding to the 3’-untranslated regions (UTRs) of target messengerRNAs (mRNAs) [1,2]. This results in repression of translation or directing the sequence-specific degradation of their target mRNAs [3]. miRNAs play a significant role in a wide range of physiological and pathological processes, including breast cancer. Furthermore they have been shown to be dysreguated in both tissue and circulation of cancer patients [4,5]. Increasing evidence implicates miRNAs in cancer progression, including tumour growth, differentiation, invasion and metastasis [6]. The miRNA of interest in this study, miR-379, is located on chromosome 14q32, 31 and to date, very little is known about its role in normal physiology. In the context of breast cancer, there is currently one report implicating miR-379 in the regulation of interleukin-11 (IL-11) production in breast cancer cell lines [7]. miR-379 has a predicted binding site on a key gene associated with breast cancer, Cyclin B1, which is known to be up-regulated and associated with poor patient outcome [8–11]. The Cyclin B1 3’ untranslated region is 612bp in length, with computational algorithms (TargetScan, miRanda) predicting a binding site for miR379 starting at position 404 [12–14].

Cyclin B1 is a key initiator of mitosis. It has a crucial role in regulating Cyclin-dependent kinase 1 (Cdk1), which initiates the progression from G2 phase to mitosis [15]. Over-expression of Cyclin B1 is associated with a number of different cancers including breast [8,11,16], oesophageal squamous cell [17,18], non-small cell lung [19,20] and renal cancer [21]. Further, over-expression of Cyclin B1 is associated with poor patient survival and increased resistance to radiotherapy in head and neck squamous cell carcinoma [22,23]. Researchers are investigating the potential of depleting Cyclin B1 expression in tumours as a therapeutic strategy, by initiating anti-proliferative and apoptosis-inducing properties [24,25]. Understanding miRNA mediated regulation of Cyclin B1 is therefore an exciting avenue of investigation. Currently two groups have shown the potential for miRNA-mediated regulation of Cyclin B1 in cancer cell lines [26,27]. The first study reported knock-down of endogenous miR-744 in a murine prostate cancer cell line which exhibited reduced Cyclin B1 expression suggesting positive regulation of the gene [26]. The second study investigated the effect of miR-494 on cell cycle progression through the G2/M phase of human cholangiocarcinoma cell lines, and found it to regulate a number of key genes involved in G2/M phase including Cyclin B1 [27].

In the present study, miR-379 expression was quantified in clinical samples which included tissues from breast cancer patients (n=103), healthy controls (n=30) and patients with benign breast disease (n=35). Any relationship with clinicopathological details was investigated. Cyclin B1 gene expression was also quantified and any association with miR-379 expression examined. The effect of miR-379 mimic on Cyclin B1 protein expression and function was investigated. Finally, the levels of circulating miR-379 were determined in patients with breast cancer (n=40) and healthy controls (n=34).

## Results

### MiR-379 expression in human breast tissues

**Figure 1:**
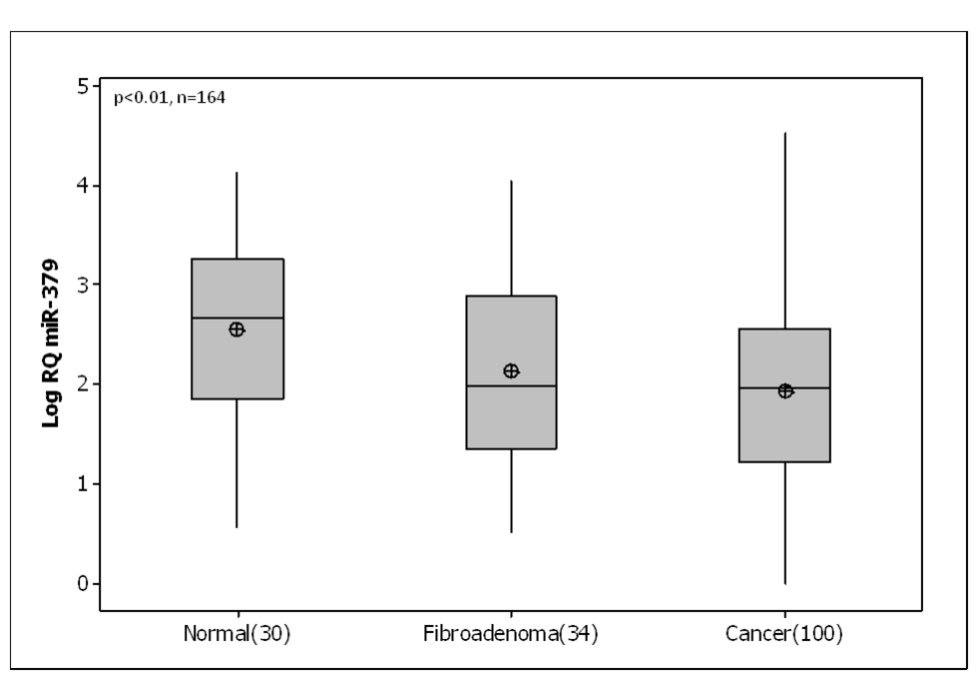
MicroRNA-379 (miR-379) expression in normal, fibroadenoma and malignant breast tissues. RQ-PCR of miR-379 revealed significantly decreased levels of expression in breast cancer n=100 (Mean(SEM) 1.9 (0.09) Log_10_ Relative Quantity) compared to normal tissue n=30 (2.6 (0.16) Log_10_ RQ, p<0.01).

**Figure 2:**
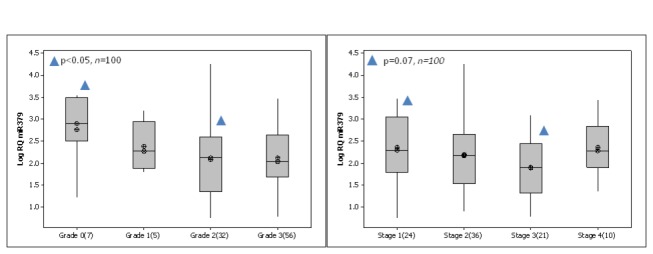
miR-379 expression across tumour stage and grade. (A) miR-379 expression across tumour stage. With increasing tumour stage, the level of miR-379 decreased significantly (p<0.05) (B) Level of miR-379 across tumour grade. A similar trend was observed for tumour grade, however it did not reach significance (p=0.128).

MicroRNA extracted from malignant (n=103), normal (n=30) and fibroadenoma (n=35) breast tissue biopsies was analysed using RQ-PCR. miR-379 was detected in 100 out of 103 breast tumours samples, and was detectable in both normal and fibroadenoma tissue. Results were expressed as Log_10_ Relative Quantity (RQ). RQ-PCR of mature miR-379 in these samples revealed a significant decrease in expression in breast tumour samples (Mean(SEM) 1.9 (0.09) Log_10_ Relative Quantity) compared to normal tissue n=30 (2.6 (0.16) Log_10_ RQ, p<0.01, Figure 1). Samples were then stratified based on patient clinicopathological details. miR-379 expression was found to show a relationship with tumour stage. With increasing tumour stage (from stage 1 to stage 3), the level of miR-379 decreased significantly (p<0.05, Figure 2A). A similar trend was observed for tumour grade however this did not reach significance (Figure 2B). There was no association of miR-379 with other clinicopathological parameters, including histological subtype (p=0.329), tumour size (p=0.243), menstrual status (p=0.486) or epithelial subtype (p=0.965, results not shown).

### Cyclin B1 expression in human breast tissues

**Figure 3:**
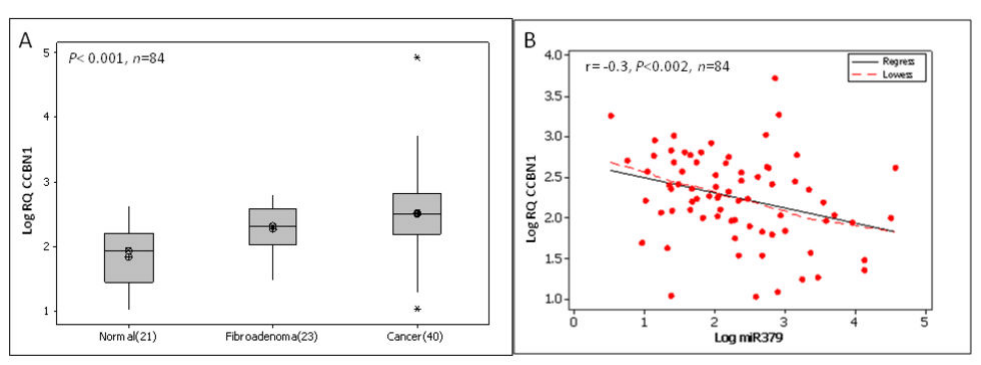
Cyclin B1 expression in normal, fibroadenoma and malignant breast tissues. (A) Cyclin B1 gene expression across all tissue types. Cyclin B1 gene expression was significantly elevated in breast cancer (Mean(SEM) 2.52 (0.10) Log_10_ RQ) compared to benign (2.15 (0.11) Log_10_ RQ, p<0.05) and normal breast tissue (1.78 (0.10) p<0.001). (B) Pearson Correlation of miR-379 and Cyclin B1. A significant negative correlation was observed between Cyclin B1 and miR-379 (r=-0.31, p<0.001).

Cyclin B1 expression was also quantified across a subset of 84 breast tissue specimens. Expression levels of Cyclin B1 were significantly elevated in breast cancer (n=40, 2.52 (0.10) Log_10_ RQ) compared to fibroadenoma (n=23, 2.15 (0.11) Log_10_ RQ, p<0.05) and normal breast tissue (n=21, 1.78 (0.10) Log_10_ RQ, p<0.001, Figure 3A). Any potential relationship between Cyclin B1 and miR-379 was then investigated. A significant negative correlation was observed between Cyclin B1 and miR-379 (r= -0.31, p<0.002, Figure 3B).

### miR-379 Effect on Cell Proliferation

**Figure 4:**
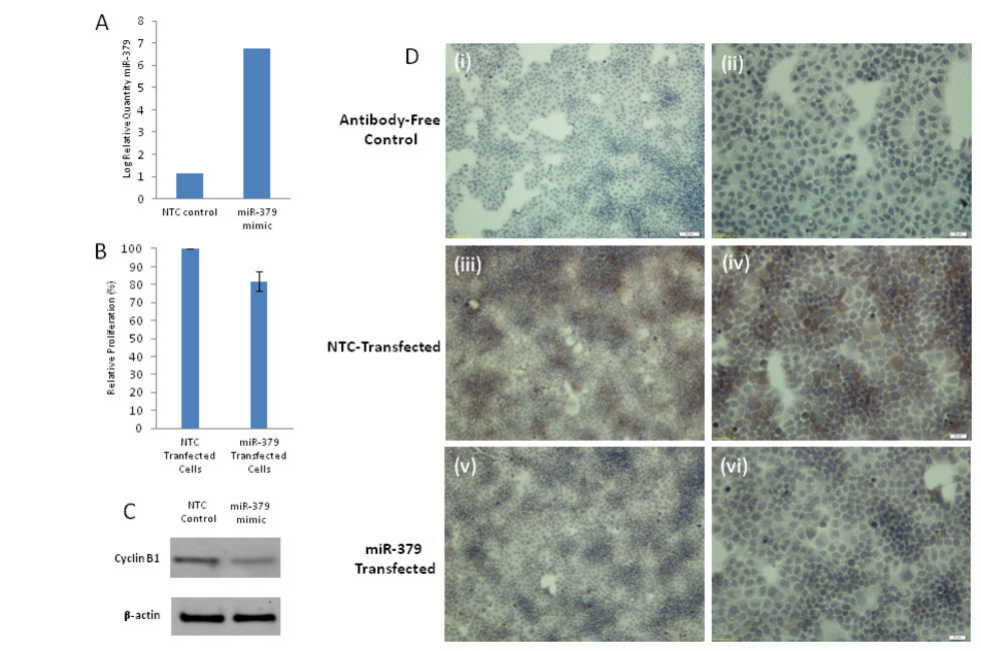
Transfection of T47D cells with miR-379 or a non-targeting control (NTC) mimic. (A) Confirmation of elevated miR-379 expression 48 hours following transfection with miR-379 mimic. (B) Cell proliferation following transfection, resulting in a decrease in proliferation (range 7-24%) in the presence of a miR-379 mimic (p<0.05). (C) The effect of miR-379 transfection on Cyclin B1 protein expression was also determined at 48 hours through western blot (C) and immunohistochemistry (D) (i, ii) antibody-free, (iii, iv) Cells transfected with NTC mimic, (v, vi) Cells transfected with miR-379 mimic for 48h, observed significantly reduced Cyclin B1 expression. Magnification (i, iii, v) 10X (50µm scale bar) and (ii, iv, vi) 20X (20µm scale bar).

T47D cells were transfected with miR-379 and NTC-mimic as described, and the change in miR-379 expression quantified by RQ-PCR (Figure 4A). A significant elevation in miR-379 (5.76 log_10_ RQ relative to T47D cells transfected with NTC mimic) was confirmed (Figure 4A) prior to analysis of cell proliferation or Cyclin B1 protein expression.

The CellTiter 96® AQ_ueous_ Non-Radioactive Cell proliferation Assay (Promega) was used to determine cell proliferation 48 hours following transfection with miR-379 in T47D cells. An inhibition of cell proliferation was observed (7-24% inhibition, p<0.05 Figure 4(B)) in cells transfected with miR-379 compared to those transfected with NTC-mimic.

### Cyclin B1 protein expression in cells transfected with miR-379

To confirm the effect of miR-379 on Cyclin B1 expression in T47D cells, cells were transfected with miR-379 or a NTC mimic, and protein extracted or cells fixed followed by western blot (Figure 4C) or immunohistochemistry (Figure 4D) respectively. Cyclin B1 protein was detected at the expected size of ~58kDa, with β-actin (loading control) detected at ~47kDa (Figure 4C). The level of immunoreactivity detected in miR-379 transfected cells was weaker than that observed when cells were transfected with the NTC mimic, with relative densitometry analysis revealing a >40% decrease in Cyclin B1 protein. This change in Cyclin B1protein expression was confirmed by immunohistochemical analysis of T47D cells fixed following transfection with miR-379 (Figure 4D). The negative control wells which did not receive an antibody, showed no staining (Figure 4D(i),(ii)). Robust native Cyclin B1 expression was detected in cells that had been transfected with NTC-mimic (Figure 4D(iii),(iv)). A significant reduction in Cyclin B1 staining was observed in cells transfected with a miR-379 mimic (Figure 4D(v),(vi)).

### Circulating miR-379

**Figure 5:**
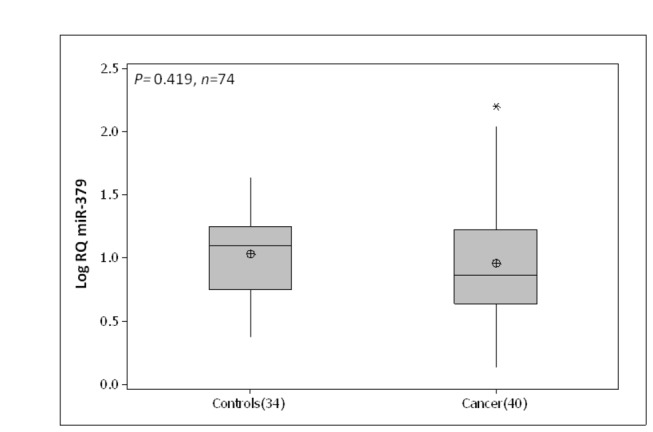
Circulating miR-379 expression across breast cancer patients and controls. No significant difference was observed between circulating miR-379 in breast cancer patients (Mean(SEM) 0.95 (0.07) Log_10_ RQ) and healthy controls (1.03 (0.05) Log_10_ RQ, p=0.42).

The circulating level of miR-379 was quantified in whole blood of 40 breast cancer patients and 34 healthy controls. No significant difference was observed between circulating miR-379 in breast cancer patients (0.95 (0.07) Log_10_ RQ) and healthy controls (1.03 (0.05) Log_10_ RQ, p=0.42, Figure 5).

## Discussion

There is no doubt that miRNAs play an important role in breast cancer, but the mode of action of many microRNAs has yet to be elucidated. Located on chromosome 14q32, 31, miR-379-410 is defined as a large cluster of brain-specific miRNAs and has been implicated in neuroblastoma as a potential biomarker [28]. While miR-379 has been shown to regulate IL-11 production in breast cancer cell lines, there have been no previous reports regarding expression levels in breast tissues. In the present study, expression of miR-379 was examined in breast tissue specimens, and was detectable in 100 out of 103 samples, and all control tissues. The level of miR-379 was significantly decreased in breast cancer compared to normal breast tissue. Due to the decreased levels of miR-379, its expression was also investigated in the breast cancer cohort for any potential relationships with patient clinicopathological details. miR-379 expression showed a significant relationship with tumour stage, with increasing tumour stage the level of miR-379 decreased significantly. A similar trend was observed with tumour grade (p=0.077). It would be worth noting that increased numbers in the Grade 1 and 2 groups may have given more significance to this aspect of the study.

miR-379 has a putative binding site on Cyclin B1 which is a key initiator of mitosis and has previously shown a relationship with tumour stage and grade in breast cancer [11]. An immunohistochemistry-based analysis of specimens from over 1,300 invasive breast cancer patients also revealed a significant association with advanced tumour stage, grade, size, oestrogen and progesterone receptor status [11]. High Cyclin B1 expression is associated with poor patient prognosis [11]. Cyclin B1 expression was investigated in a subset of breast tissue specimens (n=84), with expression levels found to be up-regulated in breast cancer compared to normal and benign breast tissue. This is in agreement with previously published work [8,11,16]. Kawamoto et al. [8] showed Cyclin B1 expression increases from benign into malignant stages of breast disease. The present study also showed a significant increase from benign to malignant breast tissues. Further, a significant increase in expression of Cyclin B1 was detected in benign compared to normal tissues. Following this investigation, a scatterplot with linear regression lines was used to assess the relationship between miR-379 and Cyclin B1, with a strong negative correlation observed, indicating a potential negative regulation of Cyclin B1. Previous studies showed knock-down of Cyclin B1 in cancer cell lines resulted in reduced cell proliferation [24,25]. Therefore potential introduction of miR-379 could be used as a therapeutic intervention to regulate the expression of Cyclin B1 and further control tumour cell proliferation. Transfection of a breast cancer cell line with a miR-379 mimic resulted in reduced Cyclin B1 protein expression and inhibition of proliferation, supporting a role for miR-379 in direct regulation of Cyclin B1.

Circulating miRNAs have the potential as biomarkers for many diseases [29]. Circulating levels of miR-379 were investigated in cancer patients compared to healthy controls. While detectable in all samples, no significant difference was observed between breast cancer patients and controls. Based on this data, it would appear that miR-379 is not a useful systemic marker of disease, but highlights a potentially important role in the primary tumour microenvironment.

The data presented identifies a role for miR-379 as a regulator of Cyclin B1 expression, with a significant loss of the miRNA observed in breast tumours.

## Materials and Methods

### Ethics Statement

All experimental procedures involving tissue samples from human participants were approved by the Clinical Research Ethics Committee (University College Hospital, Galway). Written informed consent was obtained from each patient and all clinical investigation was performed according to the principles expressed in the Declaration of Helsinki.

### Clinical Samples

Breast tissue specimens (n=168) were obtained at University College Hospital, Galway. The clinical patient samples comprised of 103 malignant tissue biopsies, 30 normal mammary tissue biopsies obtained at reduction mammoplasty, and 35 fibroadenoma tissues which are benign breast disease tissues. Full patient demographics and clinicopathological details were collected and maintained prospectively (Table 1). Samples were immersed in RNAlater® (Qiagen) for 24 hours, then the RNAlater was removed and the tissue stored at -80°C. Whole blood samples (n=74) were also collected from 40 breast cancer patients pre-operatively and 34 healthy controls with no family history of the disease. The bloods were stored at 4°C until required.

**Table 1:** Patient cohort and clinicopathological characteristics.

**Breast Clinicopathological details**	**Cancer**	**Fibroadenoma**	**Normal**
Number of patients	103	35	30
Median Patient Age yrs	56 (35-90)	44 (17-62)	46.5 (24-58)
**Menopausal Status**			
Post	72		
Pre	32		
**Histological Subtype**			
Invasive Ductal	78		
Invasive Lobular	11		
*Other*	*14*		
**Intrinsic Subtype**			
Luminal A (ER/PR+, HER2/neu-)	42		
Luminal B (ER/PR+, HER2/neu+)	18		
HER2 Over expressing (ER-, PR-, HER2/neu+)	16		
Triple-Negative (ER-, PR-, HER2/neu-)	16		
*Unknown*	*11*		
**Tumour Grade**			
1	5		
2	32		
3	55		
**Tumour size**			
1	19		
2	39		
3	10		
**UICC Stage**			
Stage 1	23		
Stage 2	36		
Stage 3	21		
Stage 4	10		

### Cell Lines and Culture Conditions

T47D breast cancer cells were previously purchased from the American Type Culture Collection (Manassas, VA). Cells were cultured in RPMI-1640 media supplemented with 10% fetal bovine serum (FBS) and 100U/ml penicillin/ 100 µg streptomycin (P/S). Cells were incubated at 37°C and 5% CO_2_ with a media change performed twice weekly.

### Total and microRNA extraction

Breast tissue specimens or cell pellets were homogenised in 1 ml TRIzol® lysis reagent (Invitrogen) as previously described [30]. Total (large and micro) RNA was extracted from malignant (n=103), normal (n=30) and fibroadenoma (n=35) mammary tissue using the RNeasy Mini Kit (QIAGEN) as per manufacturer’s instructions. MicroRNA was extracted from 1 ml of whole blood using an amended version of the TRI Reagent® BD technique (Molecular Research Center, Inc., Cincinnati, OH), as previously described [31]. Collected RNA was stored at -80°C.

### Gene and microRNA Analysis

1µg of large RNA was reverse transcribed using SuperScript III reverse transcriptase enzyme (200U/µl), 0.1 M DTT, RT-5x Buffer, RNaseOUT Ribonuclease Inhibitor (40U/µl), Random primers (3µg/µl) and dNTP’s (100mM)-Promega (Invitrogen, Carlsbad, CA, USA). TaqMan® Gene Expression Assays targeting Cyclin B1 were used in TaqMan® Universal Mastermix (Applied Biosystems). 100ng of mature microRNA was reverse transcribed using the MultiScribe™-based High-Capacity cDNA Archive Kit (dNTP 100mM, RT Buffer 10x, RNase Inhibitor 20U/µl, Stem loop primer 50nM, MultiScribe RT 50U/µl) (Appied Biosystems). The resulting cDNA for both mRNA and microRNA was analysed by ABI 79000 Fast real-time PCR system (Applied Biosystems). These reactions were carried out in a final volume of 10 µl comprising of 0.7 µl cDNA, 5 µl TaqMan® Universal PCR fast Master Mix (2x), 0.5 µl TaqMan® primer-probe mix (0.2µM), Forward primer (1.5µM), and Reverse Primer (0.7µM) (Applied Biosystems). The RQ-PCR cycle comprised of, 10-minute incubation at 95 °C followed by a 40 cycles at 95°C for 15 seconds and 60°C for 60 seconds. The use of an Inter-assay control derived from a breast cancer cell line (T47D) on each reaction allowed comparison of data across plates, and all reactions were carried out in triplicate with a standard deviation of < 0.3 considered acceptable. The relative quantity of mRNA and miRNA expression was calculated using the comparative cycle threshold (ΔΔCt). The endogenous controls used for gene expression were Mitochondrial Ribosomal Protein L19 (MRPL19) and Peptidyl-Prolyl Isomerase A (PPIA) [32]. For miRNA analysis, let-7a was the endogenous control [33], and for the blood protocol U6 was used as an endogenous control [34]. The geometric mean of the Ct value was used to normalise the data and the sample with the lowest expression level was applied as a calibrator.

### Cell Transfection

T47D cells were transfected with miR-379-5p mimic (mature sequence: UGGUAGACUAUGGAACGUAGG; 50nM) or a non-specific control miRNA (non-target control (NTC)) mimic (50nM; Switchgear Genomics, USA). Transfections were performed using Lipofectamine™ 2000 (Invitrogen, California, USA) according to manufacturer’s instructions.

### Effect of miR-379 on cell proliferation

Cells were seeded into a 96-well plate at a density of 6 x 10^4^ cells per well in antibiotic-free growth medium supplemented with 10% FBS to ensure 90-95% confluence at the time of transfection. miR-379 mimic or NTC mimic were transfected into T47D cells as described. The cells were incubated at 37°C in 5% CO_2_ and medium was changed 24 hours after transfection. Cell proliferation was measured 48 hours following transfection using CellTiter 96® AQ_ueous_ Non-Radioactive Cell proliferation Assay (Promega). As per manufacturer’s instruction, a mixture of tetrazolium compound (MTS) and phenazine methosulphate (PMS) were added to each well containing cells. The plate was incubated at 37°C and 5% CO_2_ for 3 hours before reading absorbance on plate reader (Multiskan RC, Thermo, Fisher Scientific) at 490nm.

### Effect of miR-379 on Cyclin B1 protein levels

For western blot analysis of protein expression, protein was extracted from T47D cells following transfection with the NTC mimic or miR-379 as described. Briefly, cells were washed and resuspended in Triton-X lysis buffer [150mM NaCl, 20 mM HEPES, 2 mM EDTA, 1% Triton-X100, 2mM Sodium Orthovanadate, 10mM Sodium Fluoride, 10ul/mL Protease inhibitor cocktail (Fisher Scientific)], frozen at -20°C and then centrifuged at 500 x g for 15 minutes at 4°C to remove cellular debris. The protein content was determined using the Micro BCA™ Protein Assay Kit (Thermo Scientific). Protein (100 μg) was reduced in DTT (0.5 M) for 10 minutes at 70°C and samples run on a 4-15% gradient pre-cast Mini-PROTEAN® TGX™ Gels (Bio-Rad) for 30 minutes at 200V. Protein molecular weight standards (20-220 kDa) were run simultaneously on each gel. Electroblotting was performed for 30 minutes at 100V to transfer protein samples to a nitrocellulose membrane. Blots were blocked in 5% milk in TBS-T [20 mM Tris, 137 mM NaCl, 0.1% Tween-20] for 1 hour, and probed with an antibody targeting Cyclin B1 (1:5,000; Abcam), overnight and washed in TBS-T. β-actin (1:1,000, Abcam) was used to confirm equal loading in wells. Horseradish peroxidase labelled goat anti-rabbit (1:3,000; Abcam) antibody was then added to the membranes for 1.5 hours. Following washing steps, SuperSignal West Dura Chemiluminescent substrate (Thermo Scientific) was applied to the membranes for 5 minutes. Images were captured using a Syngene G-Box and GeneSnap software. For immunohistochemical analysis of Cyclin B1 expression, cells were plated into 4-well slides (Millicell® EZ slide, Millipore) at a density of 1.75 x 10^5^ cells per well and transfected with NTC mimic or miR-379 as described. In brief, slides were put on ice for 15 minutes and then were fixed in ice-cold methanol at -20°C for 15 minutes. After methanol removal cells were incubated in 10% normal goat serum diluted in 10% PBS/0.05% Tween-20 for 30 minutes to block non-specific binding. A rabbit polyclonal antibody directed against human Cyclin B1 (1:100, ab48574, Abcam) was then applied for 90 minutes followed by washing in PBS/0.05% Tween-20. Cells were then washed and a secondary antibody tagged with HRP (1:1000, ab6721-1, Abcam) was applied for 30 minutes. After washing, detection was carried out using a peroxidise substrate kit containing the chromogen diaminobenzidine (Vector Laboratories, Burlingame, CA). Cells were then counterstained with haematoxylin for 3 minutes and the slides were dehydrated through serial alcohol immersions before mounting using Glycergel mounting medium (DAKO, Carpintera, CA).

### Statistical Analysis

All data are presented as Mean (SEM), and graphically represented using boxplots and linear scatter plots. A two sample Student’s t-test and a general model ANOVA were used to compare mean responses. Scatter plots were displayed using Linear Regression and Lowess smoother to determine the relationships between different populations. The level of relationship was determined using Pearson correlation coefficients.
